# Commentary: Safety and efficacy of remimazolam versus propofol sedation in gynecological procedures: a meta-analysis of East Asian randomized trials

**DOI:** 10.3389/fmed.2026.1821680

**Published:** 2026-06-16

**Authors:** Munder Lateiresh

**Affiliations:** Faculty of Medicine Foča, University of East Sarajevo, Foča, Bosnia and Herzegovina

**Keywords:** anesthesia, anesthesia management, gynecology, propofol, remimazolam

We read with interest the article by Jin et al. (1), which sought to evaluate the comparative safety and efficacy of remimazolam and propofol in gynecological procedural sedation. They found that remimazolam demonstrated a superior safety profile compared to propofol, with significantly lower rates of overall adverse events, respiratory depression, and hypotension. We would like to bring attention to several methodological considerations regarding data extraction, intervention classification, and multi-arm trial handling that, if addressed, could further enhance the transparency and clinical reliability of the evidence presented.

It is important to clarify that remimazolam is a short-acting benzodiazepine (2), rather than an “ultra-short” one, with a context-sensitive half-time typically ranging from 3 to 10 min, comparable to other short-acting agents. Furthermore, to provide a more comprehensive context to the available literature on remimazolam, we refer readers to a recent narrative literature review on its use in cardiac and non-cardiac surgery (3). Here, “trial-level heterogeneity” refers to variability in study designs, patient populations, and intervention protocols across the included trials, which may influence the overall treatment effect and the generalizability of the pooled findings.

In their meta-analysis, Jin et al. (1) aimed to synthesize evidence on the safety and efficacy of remimazolam vs. propofol. However, discrepancies in data extraction and outcome definition harmonization were observed, particularly concerning the study by Tan et al. (4).

Specifically, regarding hypotension, Tan et al. (4) reported a significantly lower incidence of hypotension in the remimazolam-propofol combination group (7.2%) compared with the propofol group (20.0%). However, this study was not included in the meta-analysis for hypotension [Figure 3a in Jin et al. (1)], which is a critical omission given its findings. Furthermore, Figure 3a in Jin et al. (1) reports 12 and 2 events for hypotension, but the source and context of these specific numeric events from other included studies are not explicitly detailed. This lack of clarity, coupled with the omission of relevant data from Tan et al. (4), limits the interpretability and comparability of the pooled estimate for hypotension. This highlights the importance of transparent and standardized outcome definitions and comprehensive data inclusion in safety meta-analyses.

Regarding oxygen desaturation events, Tan et al. (4) reported 12 cases of SpO_2_ < 95% in the propofol group and 2 cases in the remimazolam-propofol group (Table 4). In contrast, the meta-analysis by Jin et al. (1) reported oxygen desaturation outcomes defined using a stricter threshold (SpO_2_ < 90%). This difference in outcome definitions limits direct comparability between the trial-level and pooled data and may contribute to apparent discrepancies in event frequencies across studies. Clarification of the outcome definitions and harmonization criteria used in the meta-analysis would improve interpretability.

The classification of intervention arms warrants further discussion. The meta-analysis (1) primarily compared remimazolam monotherapy with propofol monotherapy. However, the included study by Tan et al. (4) evaluated a combination regimen consisting of remimazolam (0.125 mg/kg) plus reduced-dose propofol (1 mg/kg), compared with propofol monotherapy (2.5 mg/kg). Combining combination regimens with monotherapy-based comparisons may introduce clinical heterogeneity in the pooled analysis and may attenuate observed treatment effects and, because propofol is present in both arms of Tan et al. (4), potentially bias the pooled estimate toward the null. This issue warrants further exploration through subgroup or sensitivity analyses, for instance by excluding combination regimens or stratifying analyses according to intervention type (monotherapy vs. combination therapy). Such approaches would improve the robustness and interpretability of the pooled results.

The meta-analysis (1) included four multi-arm dose-response trials, including Zhou et al. (5), in which multiple remimazolam dosing groups were compared with a shared propofol control group. These dose arms were analyzed as independent comparisons in the pooled analysis. While this approach is methodologically acceptable when appropriately accounted for, it may introduce clinical heterogeneity related to dose variation and may increase the relative influence of individual studies.

The inclusion of multi-arm trials requires careful methodological handling to ensure appropriate estimation of variance and avoid undue influence of individual studies on pooled precision. Whether the original meta-analysis double-counted the shared propofol control group cannot be definitively determined from the reported degrees of freedom or total sample sizes; this ambiguity itself supports the need for transparent handling of multi-arm trials in future syntheses. The Cochrane Handbook recommends specific approaches such as splitting shared comparator groups or combining intervention arms where appropriate to maintain statistical validity in meta-analyses of multi-arm designs (6). This highlights the importance of transparent reporting of how multi-arm trials are incorporated into meta-analytic syntheses.

While the meta-analysis by Jin et al. (1) suggests a superior safety profile for remimazolam compared to propofol, a comprehensive clinical interpretation necessitates a thorough evaluation of the certainty of the evidence. Factors such as variations in sedation protocols, intervention dosages, and the complexity of procedures across studies can significantly impact the robustness of pooled estimates. Although statistical methods like random effects models, subgroup analyses, sensitivity analyses, and meta-regression are employed to assess these influences, the ultimate confidence in the findings hinges on a formal certainty assessment. We contend that Jin et al. (1) did not sufficiently evaluate the certainty of their findings. A statistically significant pooled estimate, if derived from studies with considerable heterogeneity or potential biases, may be prone to fragility. Therefore, applying frameworks like GRADE (Grading of Recommendations Assessment, Development and Evaluation) (7) to systematically assess the certainty of evidence is crucial. Such an assessment would provide a more nuanced understanding of the results and their implications for clinical practice, thereby strengthening the overall interpretation of the meta-analysis.

In conclusion, this commentary highlights important considerations regarding data extraction accuracy, outcome definition standardization, intervention classification, and multi-arm trial synthesis in sedation safety meta-analyses. Addressing these methodological issues may further strengthen the evidence base supporting clinical decision-making in procedural sedation. We recommend that Jin et al. (1) consider issuing a corrigendum to address the specific numeric discrepancies identified and supplement their analysis with a GRADE certainty assessment to enhance the transparency and clinical utility of their findings. Future meta-analyses may benefit from standardized reporting of sedation safety endpoints.
